# Anti-DAMP therapies for acute inflammation

**DOI:** 10.3389/fimmu.2025.1579954

**Published:** 2025-05-08

**Authors:** Russell Hollis, Megan Tenet, Monowar Aziz, Ping Wang

**Affiliations:** ^1^ Center for Immunology and Inflammation, The Feinstein Institutes for Medical Research, Manhasset, NY, United States; ^2^ Departments of Surgery and Molecular Medicine, Zucker School of Medicine at Hofstra/Northwell, Manhasset, NY, United States; ^3^ Elmezzi Graduate School of Molecular Medicine, Manhasset, NY, United States

**Keywords:** DAMPs, eCIRP, HMGB1, eNAMPT, sepsis, shock, therapeutic strategy

## Abstract

Shock, affecting a third of intensive care patients, remains a highly fatal condition despite advances in critical care, irrespective of its etiology. Cellular injury, central to shock pathophysiology, triggers the release of damage-associated molecular patterns (DAMPs), such as extracellular cold-inducible RNA-binding protein (eCIRP), high-mobility group box 1 (HMGB1), histones 3 and 4, and adenosine triphosphate (ATP). These molecules are confined within cells under normal conditions and perform essential physiological functions. However, upon their extracellular release during cellular injury, they act as alarmins, engaging pattern recognition receptors (PRRs) on immune cells. This interaction triggers a robust inflammatory response, propagating systemic inflammation and exacerbating tissue damage. Excessive DAMP-mediated inflammation is increasingly recognized as a major contributor to morbidity and mortality in a wide range of critical illnesses, including trauma, hemorrhagic shock, sepsis, and organ ischemia/reperfusion (I/R) injury. These pathologies are characterized by uncontrolled inflammatory cascades driven by the deleterious effects of DAMPs, underscoring the urgent need for targeted therapeutic interventions. This review explores the pivotal role of DAMPs in the pathogenesis of acute inflammation and shock, highlighting cutting-edge therapeutic strategies aimed at mitigating their effects. Emerging approaches include monoclonal antibodies, decoy receptors, small molecule inhibitors, and scavengers designed to neutralize or inhibit DAMP activity. The discussion also delves into the potential clinical applications of these interventions, offering insights into how targeting DAMPs could transform the management of shock and improve patient outcomes.

## 1 Introduction

Approximately one third of patients in the intensive care unit suffer from shock, a condition associated with acute inflammation and poor prognosis (1). The underlying mechanisms of shock are categorized as hypovolemic (e.g. hemorrhage), cardiogenic (e.g. myocardial function), obstructive (e.g. pulmonary embolism), and distributive (e.g. sepsis or reperfusion injury) (1–3). While etiologies vary, all forms of shock share the common feature of inadequate tissue perfusion, triggering homeostatic mechanisms to preserve vital organ function (2). Cellular injury, a key driver of shock pathophysiology, can result not only from hypoxia but also from radiation, chemical exposure, infection, trauma, and other forms of cellular damage (4). Regardless of the cause, cellular injury leads to the release of damage-associated molecular patterns (DAMPs), which amplify the inflammatory response and contribute significantly to morbidity and mortality in critically ill patients (5).

Under normal conditions, DAMPs are intracellular molecules with essential biological functions, but during cellular stress or after cell death, DAMPs are released into the extracellular space as proinflammatory molecules/mediators (6). Some DAMPs, such as extracellular cold-inducible RNA-binding protein (eCIRP), high mobility group box 1 (HMGB1), and histones are considered chromatin-associated molecular patterns (CAMPs), which are DAMPS that originate in the nucleus or are directly associated with chromatin (7). Extracellular RNA (exRNA) DAMPs consist of mostly micro and ribosomal RNA but also include messenger, long non-coding, and circular RNA (7). These nucleic acids, in addition to cell free DNA (both nuclear and mitochondrial DNA from damaged cells), are also considered CAMPs (7, 8). Other DAMPs, such as extracellular adenosine triphosphate (ATP) and heat shock proteins (HSPs), originate from the cytosol (7, 9).

DAMPs can activate immune cells and non-immune cells, such as epithelial and endothelial cells, through pattern recognition receptors (PRRs) (10). Activation of PRRs leads to cytokine and chemokine production, promoting the inflammatory response (10). While DAMPs may play a role in coordinating tissue repair and regeneration, an overly robust or uncontrolled inflammatory response leads to overwhelming systemic inflammation and consequent tissue damage, as is often evident in trauma, hemorrhage, sepsis, and autoimmune diseases (11, 12). DAMP-driven uncontrolled inflammation causes extensive tissue damage by activating cell death machineries and upregulating cytokine expression. Moreover, inflammation leads to metabolic dysfunction causing energy deficiency and mitochondrial damage, leading to cell death and tissue injury (13, 14). These sequelae can occur in the setting of acute inflammation, a response to an inciting event that lasts for days, as well as in chronic inflammation which lasts from weeks to years from repeated injury or improper healing mechanisms (15). This review will focus on acute inflammation, where DAMPS have generated increasing interest as potential therapeutic targets.

Several strategies are being developed to target DAMPs therapeutically, offering diverse mechanistic approaches. These strategies include monoclonal antibodies, decoy receptors, small molecule inhibitors, and scavenging peptides (16). Monoclonal antibodies directly neutralize specific DAMPs. Decoy receptors also bind to DAMPs, preventing their interaction with target receptors. Small molecule inhibitors interfere with intracellular signaling pathways involved in DAMP release (16). These approaches, in addition to others, are actively investigated in a range of conditions, including acute inflammatory pathologies, malignancy, neurological diseases, and autoimmune disorders.

Since the inception of Matzinger’s danger hypothesis over 30 years ago, our understanding of DAMPS continues to develop (17). Over the past 5 years, classification systems, such as CAMPS, have refined our ability to distinguish different groups of DAMPs (7). Lifestyle-associated molecular patterns (LAMPs) have emerged as a new group of DAMP-like molecules deserving investigation with the rise of obesity-related illnesses (18). LAMPS are endogenous molecules, such as cholesterol and uric acid crystals, that contribute to the progression of chronic inflammatory diseases, such as atherosclerosis and gout (18). Beyond new classification systems, novel DAMPS and DAMP mechanisms have surfaced. eCIRP, an RNA chaperone protein discovered as a DAMP in 2013 due to its activation of TLR4, was also discovered to activate triggering receptor expressed on myeloid cells-1 (TREM-1) in 2020 (19, 20). Extracellular nicotinamide phosphoribosyltransferase (eNAMPT) was discovered as a DAMP in 2015 (21, 22). NAMPT is an enzyme that regulates nicotinamide adenine dinucleotide biosynthesis (NAD) but when secreted to the extracellular space exacerbates lung injury via interaction with TLR4 (22). Extracellular receptor-interacting protein kinase 3 (RIPK3), which promotes necroptosis when intracellular, was recognized as a DAMP which activates receptor for advanced glycation end products (RAGE) in myocardial infarction and reperfusion injury in 2024 (23). The majority of therapeutics that target DAMPs or their receptors have only emerged recently, with FDA-approved medications largely on the horizon (21).

With these recent advances in mind, this review focuses specifically on the role of DAMPs in acute inflammation and shock, examining their classification, release mechanisms, inflammatory mechanisms, clinical contexts, and current therapeutic strategies.

## 2 Classification of DAMPs

DAMPs are endogenous molecules released during cellular stress that elicit inflammation through interaction with PRRs, such as TLR4 (4, 24). Activated PRRs initiate downstream signaling cascades, such as pathways involve in the translocation of nuclear factor kappa-light-chain-enhancer of activated B cells (NF-κB), which promotes cytokine production (24). Cytokines exacerbate cellular stress, creating a feed-forward loop that amplifies inflammation and further damages tissue (9). While this inflammatory cascade can be detrimental, DAMPs also play a role in tissue repair (11). For example, HMGB1 and ATP have been implicated in stem cell migration, proliferation, and the secretion of proangiogenic factors (5). This duality highlights the complex and context-dependent nature of DAMP signaling (4, 25).

Unlike cytokines, DAMPs are constitutively expressed in various cell types and have primary physiological functions distinct from their role in inflammation (9). They are also distinct from pathogen-associated molecular patterns (PAMPs), which are derived from microorganisms. DAMPs are endogenous molecules involved in both sterile inflammation and sepsis (9, 24). DAMPs are broadly classified into five molecular categories: proteins, nucleic acids, metabolites, ions, and glycans (4). Further categorization is based on cellular localization, including extracellular matrix, plasma membrane, cytoplasm, mitochondria, endoplasmic reticulum (ER), granules, and nuclear compartments (e.g., CAMPs; **Table 1**) (7, 32). This classification system provides a framework for understanding DAMP origins, release mechanisms, and receptor interactions. However, significant heterogeneity exists within each category (32). For example, while major CAMPs activate TLR4, some activate retinoic acid inducible gene I (RIG-I) or interleukin-1 receptor (IL-1R) (32). Therefore, while DAMP classification provides a helpful framework, understanding the specific functions and signaling pathways of individual DAMPs is essential.

**Table 1:** Classification of DAMPs.

DAMP Classifications	DAMPs	Functions in Homeostasis	Targets	Ref.
CAMPs/Nuclear	eCIRP	RNA chaperone protein	TLR-4, TREM-1	(7, 20)
	HMGB1	Binds to and regulates DNA	RAGE, TLRs	(7, 26)
	Histones	Stabilize chromosomes forming nucleosomes	TLR4, TLR2, TLR9, NLRP3	(7, 27)
	IL-33	Binds histones	ST2 and IL-2 heterodimeric receptor	(28)
	cfDNA	Nuclear and mitochondrial DNA from damaged cells	TLR9, cGAS, AIM-2	(7, 29)
	exRNA	Micro and ribosomal RNA	TLRs, RAGE	(7, 29)
Cytosolic	ATP	Energy source	P2X, P2Y	(30)
	HSPs	Protein chaperone	TREM-1	(31)
	eNAMPT	Involved in nicotinamide adenine dinucleotide synthesis	TLR4	(21, 22)
	RIPK3	Promotes necroptosis	RAGE	(23)

This table describes classification of the major DAMPs in acute inflammation and their respective targets. AIM-2, Absent in melanoma-2, ATP, Adenosine triphosphate, CAMPs, Chromatin-associated molecular patterns, cGAS, Cyclic guanosine monophosphate-adenosine monophosphate synthase, DAMPs, Damage-associated molecular patterns, eCIRP, extracellular cold-inducible RNA-binding protein, eNAMPT, extracellular nicotinamide phosphoribosyltransferase, HMGB1, High mobility group box 1, HSPs, Heat shock proteins, NLRP3, Nucleotide-binding domain and leucine-rich repeat protein-3, P2, Purinergic receptor 2, RAGE, Receptor for advanced glycation end products, RIPK3, Receptor-interacting serine/threonine-protein kinase 3, ST2, Soluble suppression of tumorigenicity 2, TLR, Toll-like receptor, TREM-1, Triggering receptor expressed on myeloid cells-1.

In acute inflammation, major DAMPs include eCIRP, HMGB1, histones, ATP, interleukin 33 (IL-33), eNAMPT, HSPs, RIPK3, and exDNA, and exRNA (7, 22, 23, 29). Major DAMP-sensing receptors in acute inflammation include TLR2, 4, and 9, TREM-1, RIG-I-like receptors, and RAGE (9, 32). Interactions with these receptors occur after DAMP release, which will be discussed in the following section.

## 3 Release mechanisms of DAMPs

**Figure 1:**
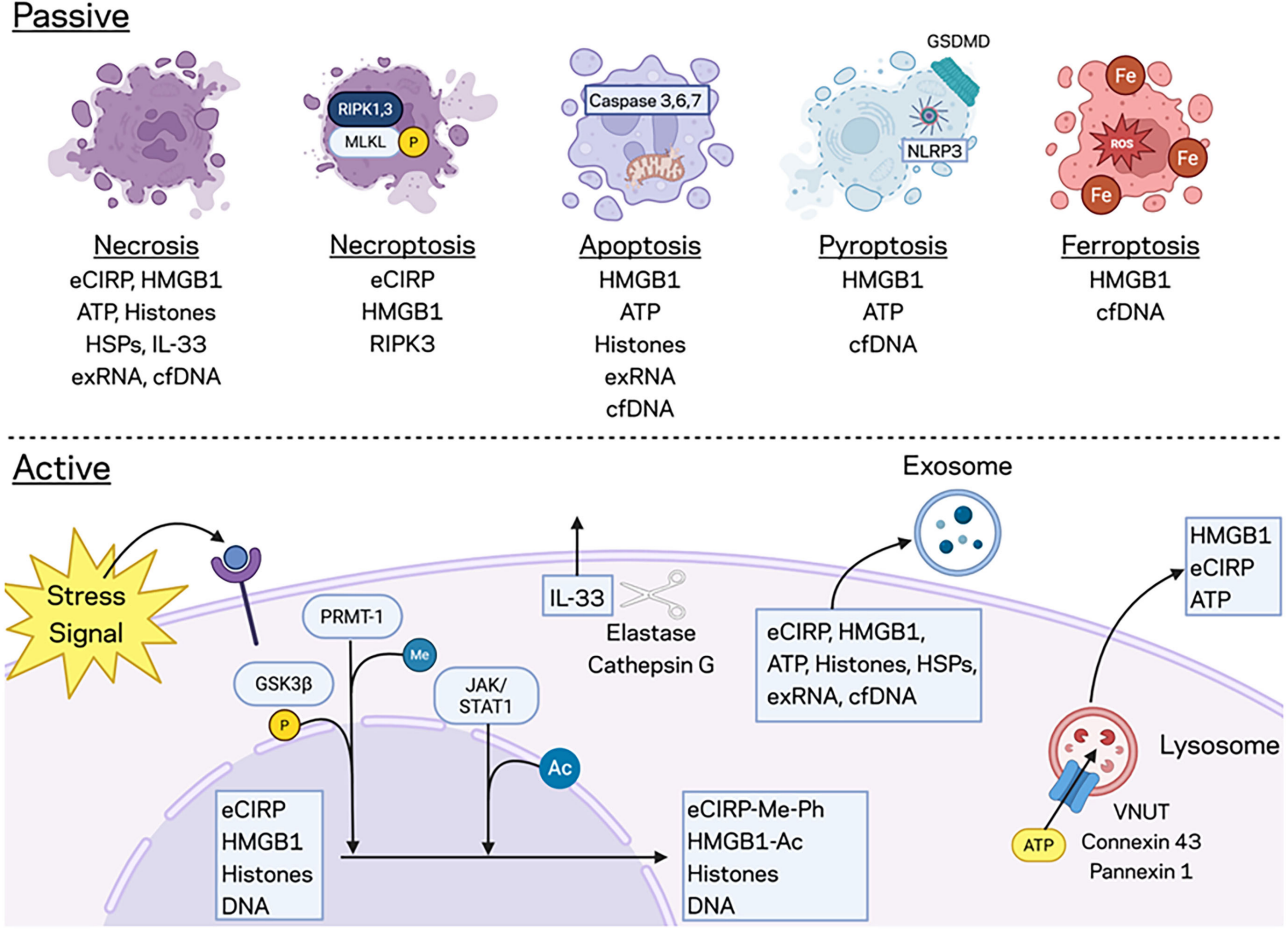
Release mechanisms of DAMPs in acute inflammation. There are several mechanisms of passive and active release for the DAMPs described in this review. Each DAMP is listed according to its known mechanisms of release. Deployment of extracellular traps by neutrophils and macrophages (not pictured) is an additional method of release. ATP, Adenosine triphosphate, cfDNA, Cell free DNA, eCIRP, Extracellular cold-inducible RNA-binding protein, exRNA, Extracellular RNA, GSDMD, Gasdermin D, GSK3 β, Glycogen synthase kinase-3β, HMGB1, High mobility group box 1, HSPs, Heat shock proteins, IL-33, Interleukin 33, JAK, Janus kinase, MLKL, Mixed lineage kinase domain-like pseudo kinase, NLRP3, Nucleotide-binding domain, leucine-rich repeat, and pyrin domain-containing protein 3, PRMT-1, Protein arginine methyltransferase 1, RIPK, Receptor-interacting protein kinase, ROS, Reactive oxygen species, STAT-1, Signal transducer and activator of transcription 1, VNUT, Vesicular nucleotide transporter.

DAMPs are released through active and passive mechanisms during cellular injury or stress (**Figure 1**). Some release mechanisms, such as extracellular trap formation (ETosis), can be either active or passive depending on the context (29). In active release, CAMPs, such as eCIRP, histones, and HMGB1 require translocation from the nucleus to the cytoplasm (7). Transportation across compartments often requires post-translational modifications, such as methylation, phosphorylation, or acetylation (4, 9, 29). DAMP activity, either pro or anti-inflammatory, can be different depending on cell types, including immune on non-immune cells, as well as post-translational modifications (33–35). This review will focus on the proinflammatory activities of DAMPs.

### 3.1 Passive release

Passive release is attributed to several forms of cell death, such as necrosis, necroptosis, apoptosis, pyroptosis, and ferroptosis as well as production of extracellular traps (29). Necrosis is pathological, unorganized cell death that releases intracellular contents suddenly into the extracellular space, specifically eCIRP, HMGB1, ATP, histones, HSPs, IL-33, extracellular RNA (exRNA), and cell free (cf) DNA (9, 29, 36). Necroptosis is a more organized form of cell death that releases eCIRP, HMGB1, and RIPK3 (23, 37, 38). Necroptosis requires activation of mixed lineage kinase domain-like pseudo kinase (MLKL) through phosphorylation by receptor-interacting protein kinases 1 and 3 (RIPK 1/3). Activated MLKL forms a pore in the cell-membrane that allows for the release of DAMPs (37).

In apoptosis, intrinsic or extrinsic signals promote caspase-3, -6, and -7 activity, leading to chromatin condensation, DNA fragmentation, and membrane blebbing (29). Apoptosis releases HMGB1, ATP, histones, exRNA, and cell free (cf) DNA (29). For example, histone release is specifically associated with DNA fragmentation, and CAMPs in general are transported to the cell membrane for release (29). ATP can also be released through apoptosis via protein kinase R-like endoplasmic reticulum kinase (PERK) activity (29). While these DAMPs exacerbate inflammation during apoptosis, HMGB1 adopts an immunosuppressive phenotype through terminal oxidation of its cysteine residue C106 during this organized form of cell death (34, 35, 39, 40). Although apoptosis is primarily considered to passively release DAMPs, programmed release of exosomes with eCIRP can be interpreted as active (41).

Pyroptosis results from induced gasdermin D pore formation by caspase-1 after activation of the nucleotide-binding domain and leucine-rich repeat protein-3 (NLRP3) inflammasome or caspase-4, -5, and -11 (4, 29). DAMPs such as HMGB1, ATP, and cfDNA are small enough to leak though these pores into the extracellular space, although HMGB1 release most likely occurs after the cell lysis stage of this process (4, 9, 42, 43).

Ferroptosis results from intracellular iron stores that promote the production of radical oxygen species (ROS), which leads to oxidative stress. HMGB1, after acetylation, is released along with cfDNA in ferroptosis (29).

### 3.2 Active release

For active release, a stress signal is required (29, 44). Conventional active release depends on signal peptides that trigger ER-Golgi apparatus packaging and secretion via vesicles (29). Many DAMPs do not have signal sequences and are therefore actively released by non-conventional manners in exosomes (eCIRP, HMBG1, ATP, histones, HSPs, exRNA, and cfDNA) or lysosomes (HMGB1, eCIRP, and ATP) (9, 29, 45, 46). Lysosomal and exosomal secretion are achieved through fusion to the cell membrane mediated by Ras-associated binding protein (RAB) and Soluble N-ethylmaleimide-sensitive factor activating protein receptor (SNARE) complexes (29) (9, 46).

For HMGB1, oxidative stress, ionized calcium, cytokine release, or other activation of macrophages leads to nuclear translocation, which requires acetylation (44). Acetylation is mediated by the Janus kinase/signal transducer and activator of transcription 1 (JAK/STAT-1) pathway and is performed by histone acetyltransferase proteins on nuclear localization sequences 1 and 2 (NLS1 and NLS2) (44, 45). Acetylation is followed by further modifications, like methylation (specifically at Lys42 to reduce DNA-binding), phosphorylation, and poly(ADP)-ribosylation, which designate HMGB1 for secretion (45). HMGB1 can also be released via the inflammasome through ATP-dependent stimulation of the purinergic receptor P2X7, which activates the p38/Mitogen-activated protein kinases (MAPK)/NF-kB pathway (29). Unlike the immediate release observed during necrosis, active HMGB1 secretion requires a longer timeframe (24).

Active eCIRP release is triggered by various cellular stressors, including endotoxin challenge, oxidative stress, osmotic stress, heat shock, and ER stress (47). Nuclear translocation to stress granules requires methylation by protein arginine methyltransferase 1 (PRMT-1) and phosphorylation by glycogen synthase kinase-3β (GSK3β) and casein kinase II (CK2) (29, 41, 47). Formation of the stress granule is regulated by T-cell intracellular antigen 1 (TIA-1), fragile-X mental retardation protein (FMRP), cytoplasmic polyadenylation element Th-binding protein 1 (CPEB1), and TIA-1 related/like protein (TIAR) (41, 47). These eCIRP-containing stress granules are subsequently packaged into exosomes and released from the cell (29).

For other DAMPs, the mechanisms of release are not as well known. Translocation of histones 3 and 4 can be triggered by hypoxia or pathogenic invasion (48, 49). Different stresses can increase release of ATP, but hypoxia has been found to reduce its release (30). Vesicular nucleotide transporter (VNUT) protein, facilitated by a proton gradient, stores ATP into lysosomes for secretion (29, 30). Additionally, ATP can transport through connexin 43 or pannexin 1, which open during electrolyte imbalances (29, 30). HSPs are found in exosomes and ectosomes, outward buddings that form in response to elevated intracellular calcium (29). IL-33’s release is downregulated by oxidation but upregulated by other post-translational modifications, such as cleavage by neutrophil elastase and cathepsin G, maintaining its IL-1-like domain, which enhances its pro-inflammatory activities (50).

## 4 Mechanisms of DAMP-induced inflammation

After release, DAMPs interact with multiple receptors to stimulate inflammation, but they do not all act on the same receptors. The inflammatory mechanisms of DAMPs will be discussed below by receptor interaction.

### 4.1 Toll-like receptors

Multiple TLRs interact with DAMPs to promote inflammation, including TLR2, TLR4, and TLR9. TLR2 is a receptor for histones, and TLR4 is a major receptor for eCIRP, HMGB1, histones and eNAMPT (4, 22). Both TLR2 and TLR4 activate cyclic AMP response element binding protein (CREB) and activator protein-1 (AP-1) via myeloid differentiation primary response protein 88 (MyD88)/MAPK as well as NF-kB via the MyD88/IκB kinase (IKK) complex. These three pathways promote mRNA transcription of pro-inflammatory cytokines (4). Additionally, TLR4 activates interferon regulatory factor 3 (IRF3) via the TIR-domain-containing adapter-inducing interferon-β/stimulator of interferon genes/tank-binding kinase 1 (TRIF/STING/TBK1) pathway to produce interferons (51). TLR4 also upregulates NADPH oxidase activity when stimulated by HMGB1 (33). While TLR9 is on the inner surface of endosomes, the receptor is activated by the influx of histones and HMGB1 (26, 52).

### 4.2 Triggering receptor expressed on myeloid cells-1

TREM-1 is a DAMP receptor due to its interactions with eCIRP, HMGB1, and HSPs (24, 31, 53, 54). TREM-1 regulates the activity of numerous downstream proteins, starting with formation of the TREM-1/DNAX activating protein of 12 kD complex (DAP12) (31). Spleen tyrosine kinase (SYK) then binds to a phosphorylated DAP12, ultimately leading to activation of CREB, AP-1, and NF-kB through various signaling pathways, such as phosphatidylinositol 3-kinase/protein kinase B (PI3K/AKT) (55). These transcription factors are associated with increasing production of several cytokines and chemokines, including IL-1β, IL-6, and tumor necrosis factor alpha (TNF-α) (55).

### 4.3 Receptor for advanced glycation end products

RAGE is a receptor to RIPK3 and HMGB1 (9, 23). Its expression increases in disease states, specifically in endothelial cells and leukocytes, whereas basal expression is low in most tissues (56). RIPK3 binds to RAGE to activate Ca^2+^/calmodulin-dependent kinase II (CaMKII), which increases cytokine production (23). HMGB1 binds RAGE to activate NF-kB via multiple pathways, including TRIF/MAPK and PI3K/AKT, particularly on monocytes and macrophages (9, 33). In addition, RAGE induction can lead to increased expression of cellular adhesions molecules, such as vascular cellular adhesion molecule 1 (VCAM-1), intracellular adhesion molecule 1 (ICAM-1), and C-X-C motif chemokine ligand 12 (CXCL12) (9, 25). However, there is also evidence the HMGB1 interaction with RAGE decreases NADPH oxidase activity of neutrophils (33).

### 4.4 NLRP3

NLRP3 activity, which releases DAMPs, can also be stimulated by DAMPs, such as the CAMPs eCIRP, histone 3, and histone 4, during oxidative stress (4, 7, 27). While NLRP3 is intracellular rather than at the cell-surface, it is stimulated by the influx of DAMPs into the cytosol (27, 57). Some evidence indicates that NLRP3 is activated by ROS in a TLR9-dependent manner after TLR9 is stimulated by extracellular histones (52). Activation of NLRP3 leads to IL-1β and IL-18 production and release via deglutathionylation of apoptosis-associated speck-like protein (ASC) and cleavage of caspase-1 (27, 58, 59).

### 4.5 Suppression of tumorigenicity 2

ST2 is the main target of IL-33 (9). ST2 belongs to the IL-1 receptor (IL-1R) family and is expressed on Th2 cells and mast cells. IL-33 binds to ST2, which forms a complex with IL-1R3 and promotes the MyD88-interleukin-1 receptor-associated kinase (IRAK)-dependent activation of NF-kB, c-Jun N-terminal kinase (JNK), and MAPK (45). This in turn increases expression of several pro-inflammatory chemokines and cytokines. IL-33 and ST2 interactions also contribute to Th2 cell maturation (45). Oxidation of IL-33 or the interaction with soluble ST2 (sST2) decoys can inhibit this process.

### 4.6 Purinergic 2 receptors

P2 family receptors are the primary target of ATP as a DAMP (4). In addition, biglycan can bind to P2 receptors, specifically P2X7, to activate the NLRP3 inflammasome downstream (4, 60). After loss of one phosphate group in ATP, ADP can bind to P2Y12R to activate NF-kB and MAPK (61).

### 4.7 Cyclic guanosine monophosphate-adenosine monophosphate synthase

cGAS is a demonstrated receptor of nucleic acid-based DAMPs, neutrophil extracellular traps (NETs), and HMGB1 (4). Like TLR9, cGAS is intracellular, but it is located within the cytoplasm (26). cGAS is activated by HMGB1 to produce type I interferons via STING/IRF3 (4).

## 5 DAMPs in acute inflammatory diseases

DAMPs exacerbate many acute inflammatory diseases that progress to shock. In this section we summarize several acute, inflammatory pathologies that demonstrate elevated levels of DAMPs in humans. Many of these pathologies naturally progress to shock, a phenomenon associated with DAMP-related immune dysregulation (17). Most studies measuring DAMPs in humans evaluate serum levels by ELISA, but there are studies that have measured DAMPS in stool samples, bronchoalveolar lavage, and tissue biopsies, such as in the colon (5, 62–73).

### 5.1 Sepsis

Sepsis progresses from multiple infections, such as pneumonia and intrabdominal sources. Among patients in the intensive care unit (ICU) with a diagnosis of sepsis, higher levels of CIRP were found in the serum of non-survivors compared to survivors, with a 96.77% sensitivity at 1.47ng/ml (74). Serum levels of HMGB1 have also been compared in septic patients and healthy controls, with higher concentrations of HMGB1 observed in septic individuals, yielding a sensitivity of 75.8% at 10.45 pg/ml (75). HMGB1 is also correlated with mortality and heart dysfunction in sepsis (68, 76, 77). Histones were found to be elevated in septic patients and associated with the need for renal replacement, elevated troponin levels, and mortality (65, 67, 78). Studies regarding ATP levels in the plasma of septic patients are mixed, showing both increases and decreases when compared to controls, perhaps due to rapid degradation (79, 80). High IL-33 levels were associated with survival for adults in a study evaluating patients in the medical ICU (64).

### 5.2 Ischemia/reperfusion

I/R can occur in organ transplantation, where ischemia occurs during procurement, and reperfusion occurs upon transplantation, releasing DAMPs. For example, deceased donor kidneys have increased renal tubule levels of TLR4 and HMGB1 after ischemic periods (81). Extracellular histones are elevated in humans after liver transplantation. Serum histones peaked in the first 24 hours, and higher levels were associated with graft rejection as well as elevated cytokine levels (82). A separate study also indicated higher levels of IL-33 in liver transplant patients who experienced postreperfusion syndrome (83). In addition to organ transplantation, I/R also occurs in thromboembolic disease, such as ischemic stroke and mesenteric ischemia, after circulation is restored. In stroke patients, serum HMGB1 increased after thrombolytic-induced reperfusion, with serum concentration increasing as infarct size increases (84).

### 5.3 Acute pancreatitis

Acute pancreatitis, a disease most often caused by obstructing biliary stones or excessive alcohol use, has shown evidence of HMGB1 release by necrotic pancreatic acinar cells (85). HMGB1 and soluble RAGE (sRAGE), a product of RAGE activation, are elevated in patients hospitalized for acute pancreatitis, with increasing elevation attributed to increasing severity of disease (68, 86). HMGB1 especially has a high specificity (100%) and sensitivity (93%) for acute pancreatitis at 0.84 ng/ml (68). In turn, cytokine levels IL-6 and IL-1β are elevated in acute pancreatitis and associated with disease severity (85). IL-18 also has an observed increase in the serum, with an even greater increase in the peritoneal fluid (85).

### 5.4 Trauma and hemorrhagic shock

Traumatic injuries and associated hemorrhage can also lead to a dysregulated systemic immune response with increased levels of DAMPs. Serum histone levels are elevated in patients suffering from multiple traumatic injuries (78). HMGB1 increases in the serum of trauma patients and does not always decrease with resolution of the traumatic injury (87–89). Patients with blunt trauma to the chest are more likely to have elevated serum HMGB1 (62). Serum levels of IL-33 are also increased acutely in severely injured trauma patients compared to healthy controls (90). Serum from patients in the surgical ICU with hypotension from trauma and/or hemorrhagic shock have also been collected and evaluated for CIRP levels. The CIRP levels in these patients were significantly higher than those of healthy controls (20).

### 5.5 Acute respiratory distress syndrome

Pathologies that lead to shock can cause acute lung injury (ALI) that progresses to ARDS, a potentially fatal condition of critically ill patients with associated elevation of DAMPs (63). Extracellular histones have been implicated, with higher levels observed in bronchoalveolar fluid (BALF) during the first 10 days of ARDS compared to an absence of histones in the BALF of healthy controls (63, 71). In addition, serum levels of sRAGE were correlated with an inability to clear alveolar fluid in ARDS (72). While HMGB1 levels were not measured in this study, this may imply a role for this DAMP given its relationship with sRAGE. Furthermore, NAMPT, also known as Pre-B-cell colony-enhancing factor (PBEF), is elevated in the BALF and serum of patients suffering from ALI and ARDS compared to healthy controls (91). Within cohorts of patients with ARDS, lower serum NAMPT levels were associated with survivorship (73).

### 5.6 Pediatric disease

In addition to the evidence associating DAMPs with these acute pathologies in adults, there are some studies that evaluate DAMPS in pediatric patients. In childhood sepsis, there is a significant increase in serum IL-33 levels observed from the first day of sepsis compared to control samples (92). These results differed from the previously mentioned adult studies, where high IL-33 levels predicted survivorship (64). HMGB1 levels in serum were correlated with indicators of disseminated intravascular coagulation (DIC) and severity of disease in pediatric trauma (93). Another study found significantly elevated serum HMGB1 in necrotizing enterocolitis patients, which correlated with increased levels of cytokines (66). eCIRP is elevated in the stool of neonates with necrotizing enterocolitis (70).

## 6 Therapeutics strategies to inhibit DAMPs

Several major DAMPs, such as eCIRP, HMGB1, histones, ATP, IL-33, eNAMPT, and RIPK3 are elevated during sepsis, hemorrhagic shock, I/R, acute pancreatitis, traumatic injury, ARDS, and pediatric diseases. The presence of DAMPs in these conditions has inspired efforts toward generating anti-DAMP therapies. Strategies to neutralize DAMPs fall into two main categories: direct and indirect inhibition. Direct inhibition strategies include neutralizing antibodies, miRNA mimics, decoy receptors, and scavengers. Indirect inhibition targets DAMP signaling pathways by blocking DAMP receptors or preventing DAMP release. The following subsections will detail current therapeutic strategies.

### 6.1 eCIRP

**Figure 2:**
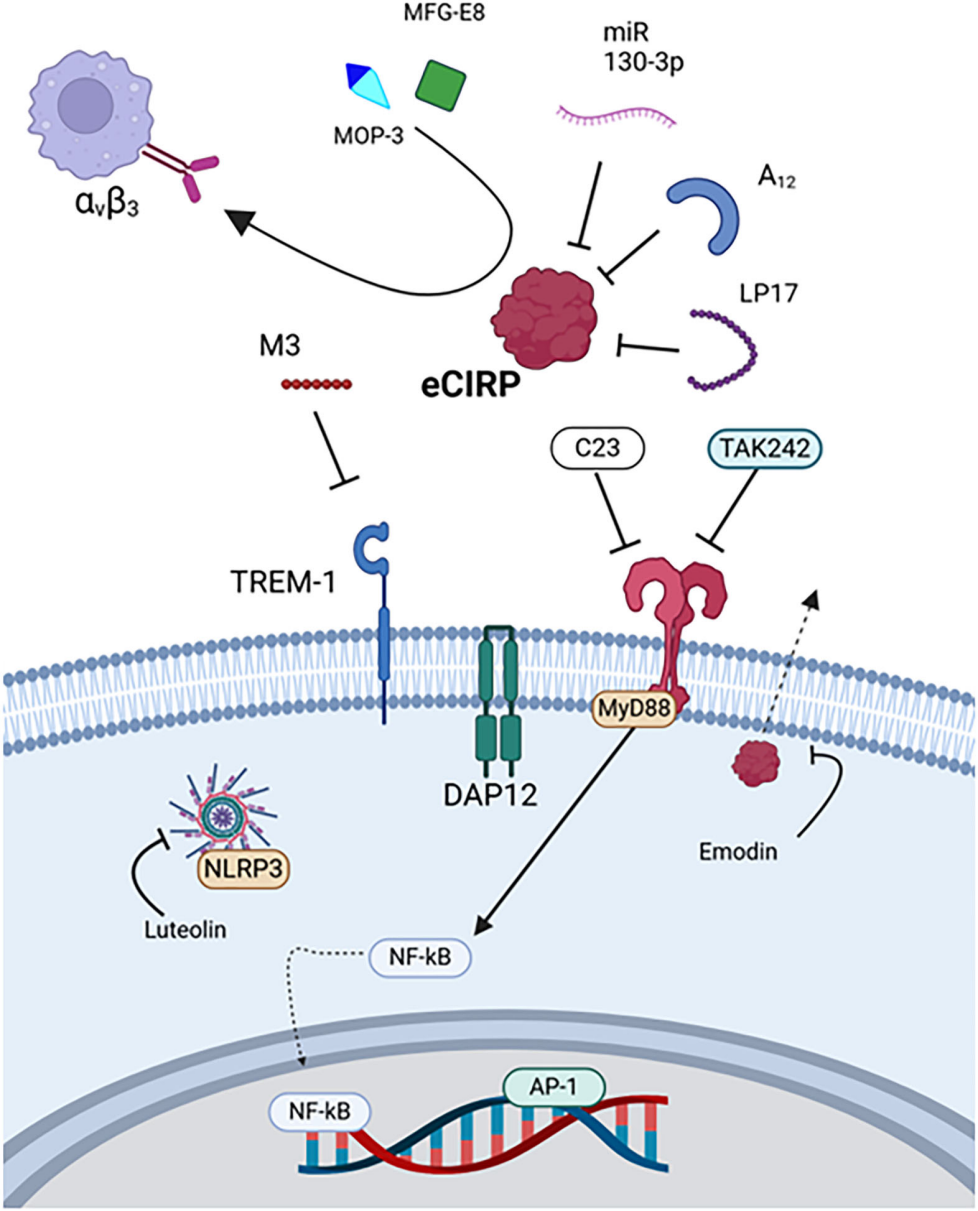
Therapies targeting eCIRP. eCIRP is targeted by MOP3, MFG-E8, miR-130-3p, A_12_, and LP17. Its receptor TREM-1 is targeted by M3, and TLR4 is targeted by C23 and TAK242. Luteolin downregulates inflammasome activity, and emodin prevents eCIRP release. eCIRP, Extracellular cold-inducible RNA-binding protein, MAPK, Mitogen-activated protein kinase, MFG-E8, Milk fat globule-epidermal growth factor VIII, MOP3, Milk fat globule-epidermal growth factor VIII-derived oligopeptide 3, MyD88, Myeloid differentiation primary response protein 88, NF-κB, Nuclear factor kappa-light-chain-enhancer of activated B cells, TLR4, Toll-like receptor 4, TREM1, Triggering receptor expressed on myeloid cells-1.

CIRP ordinarily is an RNA chaperone protein that resides in the nucleus and may be translocated to the cytoplasm (20). During cellular stress or death, CIRP is released, becoming eCIRP, a relatively novel and potent DAMP (29). eCIRP is elevated in humans after shock and has been studied in animal models of sepsis, trauma, hemorrhagic shock, and ischemia/reperfusion injury, including stroke (19, 20, 24, 74, 94–104). The 18-kDA protein promotes inflammation through activating TLR4 and TREM-1 receptors (19, 20). Several therapeutic agents with varying mechanisms have been proposed and evaluated in these preclinical models (**Figure 2**).

eCIRP was first discovered as a DAMP in a rat hemorrhagic shock model, where rats were treated with anti-CIRP antibody (**Table 2**) (20). The anti-CIRP antibody treated rats had decreased cytokine levels and organ injury markers in addition to improved survival. These results were also observed in CIRP knockout rats subjected to hemorrhagic shock and septic rats treated with anti-CIRP antibody in a cecal ligation and puncture (CLP) model (20).

**Table 2:** DAMP Therapeutic Strategies in Acute Inflammation.

DAMPs	Direct/ Indirect	Therapy	Mechanism	Pathways Involved	Ref.
eCIRP	Direct	anti-CIRP ab	Binds eCIRP	TLR4	(20)
		miR 130-3p	Binds eCIRP	TLR4/NF-κB	(98)
		PS-OMe mir130-3p	Binds eCIRP	TLR4/NF-κB	(99, 100, 102)
		A_12_	Binds eCIRP	TLR4/NF-κB	(105, 106)
		MFG-E8	Binds eCIRP and signals uptake through α_v_β_3_ integrin	TLR4,TREM-1	(94)
		MOP3	Binds eCIRP and signals uptake through α_v_β_3_ integrin	TLR4, TREM-1, α_v_β_3_ integrin	(70, 94, 104, 107, 108)
		LP17	TREM-1 decoy	TREM-1/DAP12	(19, 109)
	Indirect	C23	Binds TLR4	TLR4/NF-κB	(103, 110, 111)
		TAK242	Binds TLR4	TLR4/MyD88/ NF-κB	(112)
		M3	Binds TREM-1	TREM-1/DAP12	(19, 101, 113)
		Emodin	Rhubarb root derivative, inhibits eCIRP expression/release	TLR4/NF-κB, NLRP3	(114)
		Luteolin	Decreases HIF-1α and NLRP3 expression	eCIRP production	(115, 116)
HMGB1	Direct	h2g7 ab	Binds HMGB1	TLR4, RAGE/ NF-κB	(112)
		sRAGE	Binds HMGB1 to inhibit interaction with RAGE	RAGE/NF-κB	(112)
		Glycyrrhizin	Binds HMGB1	HMGB1 translocation	(117–120)
		rCD5L	Binds HMGB1 to facilitate clearance	RAGE	(121)
	Indirect				
		TLR4 ab	Binds TLR4 to inhibit interaction	TLR4/SOCS3/mTOR	(122)
		Eritoran	LPS derivative, binds TLR4/MD2 complex	TLR4/MD2/ NF-κB	(123)
		Ethyl pyruvate	Ester derivative, regulates MAPK and Nrf2	HMGB1 release, RAGE expression	(112)
		Luteolin	Flavonoid, targets syk, src, and SOCS3	STAT3/IRF-1, MyD88/NF-κB, TRIF/AP-1	(115, 124)
		Diflusinal	Derivative of salicylic acid, binds HMGB1-CXCL12	HMGB1-CXCL12 heterocomplex	(112, 125)
		Quercetin	Flavonoid, IκBα preservation, inhibits p65 nuclear translocation, phosphorylation of p38, c-Jun, and NH_2-_terminal kinase	NF-κB, MAPK	(126)
		Pyrrolidine dithiocarbamate	Inhibits NF-κB p65 nuclear translocation	NF-κB	(127)
		Edaravone	IκBα recovery, inhibits iNOS expression	NF-κB	(128)
Histones	Direct	Anti-Histone (3,4) ab	Binds histones (3,4)	TLR4, NLRP3	(65, 129)
		Activated protein C	Cleaves histones	NLRP3	(129)
ATP	Direct	Apyrase	Metabolizes ATP	P2X7, NLRP3 inflammasome	(130, 131)
		CD39	Metabolizes ATP	P2X7, NLRP3 inflammasome	(131)
	Indirect	Suramin	P2 receptor antagonist that inhibits interaction with ATP	P2X7	(130)
		A438079	P2X7 receptor antagonist	P2X7	(132)
IL-33					
	Direct	sST2	Binds IL-33	ST2L, TLR4/MyD88/ NF-κB	(130, 133)
	Indirect	Sesamin (also for HMGB1)	Sesame oil derivative, reduces JNK phosphorylation	HMGB1/TLR4/ IL-33	(134)
eNAMPT	Direct	ALT-100 mAb	Binds eNAMPT	TLR4/NF-κB	(21, 135)
		C269 mab	Binds eNAMPT	TLR4/NF-κB	(136)

This table describes therapies for the DAMPs listed, their proposed mechanisms of action, and inflammatory pathways involved. AP-1, Activator protein-1, ATP, Adenosine triphosphate, CXCL 12, C-X-C motif chemokine ligand 12, DAMP, Damage-associated molecular pattern, DAP12, DNAX activating protein of 12 kD, eCIRP, extracellular cold-inducible RNA-binding protein, eNAMPT, extracellular nicotinamide phosphoribosyltransferase, HMGB1, High mobility group box 1, iNOS, inducible nitric oxide synthase, JNK, c-Jun N-terminal kinase, LPS, Lipopolysaccharide, MAPK, Mitogen-activated protein kinase, MD2, Myeloid differentiation protein 2, MFG-E8, milk fat globule-epidermal growth factor VIII, mTOR, Mammalian target of rapamycin, MyD88, Myeloid differentiation primary response protein 88, NF-κB, Nuclear factor kappa-light-chain-enhancer of activated B cells, NLRP3, Nucleotide-binding domain, leucine-rich repeat, and pyrin domain, Nrf2, nuclear factor erythroid 2-related factor 2, P2X7, Purinergic receptor 2X7, RAGE, Receptor for advanced glycation end products, SOCS3, Suppressor of cytokine synthesis 3, Src, proto-oncogene tyrosine-protein kinase, ST2L, Suppression of tumorigenicity 2, Syk, spleen tyrosine kinase, TLR4, Toll-like receptor 4, TREM-1, Triggering receptor expressed on myeloid cells-1, TRIF, TIR-domain-containing adapter-inducing interferon-β.

Therapeutics utilizing miRNA and miRNA mimics have also been developed (98, 99, 102, 105, 106, 137). For example, a recombinant form of miR 130-3p was developed to bind eCIRP, which prevented TLR4 activation, decreasing cytokine expression in mice (98). Later, the miRNA mimic was modified with the addition of phosphorothioate O-methyl groups to improve stability (99). In addition, a modified poly-adenosine (poly(A)) tail, A_12_, was derived from the miRNA transcription processes and discovered to have high affinity with eCIRP (105, 106). These three therapeutics, miR 130-3p, modified miR 130-3p, and A_12_, all effectively reduced cell-death and cytokine expression as well as survival in mouse models of sepsis and I/R (98, 99, 102, 105, 106, 137).

Scavenger molecules represent a distinct class of DAMP-targeting therapeutics. While they share the common feature of DAMP binding with other direct inhibition strategies, scavengers uniquely promote DAMP clearance through mechanisms like phagocytosis and endocytosis (70, 94, 104, 107, 108). Milk fat globule-epidermal growth factor- VIII (MFG-E8) is an endogenously produced glycoprotein that scavenges apoptotic cells and was found to bind and scavenge eCIRP as well (94). This glycoprotein may represent one mechanism through which the body naturally eliminates DAMPs and has reduced inflammation in murine sepsis when administered as a treatment (94). However, the size and complexity of MFG-E8 posed challenges for therapeutic development. Consequently, a smaller, high-affinity derivative peptide, MOP3, was designed (94). MOP3 binds to eCIRP and interacts with α_v_β_3_ integrin via its arginylglycylaspartic acid (RGD) sequence to facilitate phagocytosis by macrophages and intestinal epithelial cells, reducing injury and improving survival in sepsis, neonatal sepsis, necrotizing enterocolitis, and I/R models (70, 94, 104, 107, 108).

An example of indirect inhibition is C23, a peptide derived from the region of eCIRP that binds TLR4. The oligopeptide has a higher affinity with the MD2 portion of the TLR4/MD2 complex (*K_D_* =2.97×10^–8^ M) than eCIRP and can reduce inflammation and injury in mice subjected to sepsis, hemorrhagic shock, mesenteric I/R, or pancreatitis (103, 110, 111, 138). TAK242 is a small molecule TLR4 inhibitor that ameliorates inflammation induced by eCIRP in rat models of acute pancreatitis with associated lung injury (114). Another example of indirect inhibition, M3, is a peptide derived from the region of eCIRP that binds TREM-1 (19, 101, 113, 139). In studies evaluating the role of eCIRP’s interaction with TREM-1, reductions in cytokine expression, lung injury, and mortality were observed in septic mice or hemorrhagic rats when treated with M3 or the TREM-1 inhibitor LP17, respectively (19, 109, 139). M3 was also studied in mice subjected to mesenteric I/R and showed inhibition of eCIRP’s damaging effects (113).

Naturally occurring compounds, such as emodin and luteolin have been explored as well (115, 116, 124, 140). Emodin (1,3,8-trihydroxy-6-methylanthraquinone) is a small, organic compound isolated from rhubarb root. This compound showed efficacy in a rat model of acute pancreatitis and associated lung injury as well as eCIRP stimulation *in vitro* (114). In these scenarios, emodin inhibited NF-kB and NLRP3 inflammasome-mediated inflammation downstream of TLR4 activation (114). Luteolin (3’, 4’, 5, 7-tetrahydroxyflavone), which can be isolated from a variety of plants, reduced serum eCIRP expression, inflammation, and lung injury in mice subjected to sepsis by cecal slurry (116).

### 6.2 HMGB1

HMGB1 is a member of the high-mobility group proteins, which are non-histone DNA-binding proteins with a variety of biological functions (141). HMGB1 is the most common of this family of proteins, localized mainly in the nucleus, though it travels from the nucleus to the cytoplasm of all cells. Under resting conditions HMGB1 plays an important role in regulation of chromatin structure and gene expression (45). Once in extracellular space, HMGB1 interacts with several receptors, most commonly RAGE and TLRs (4).

**Figure 3:**
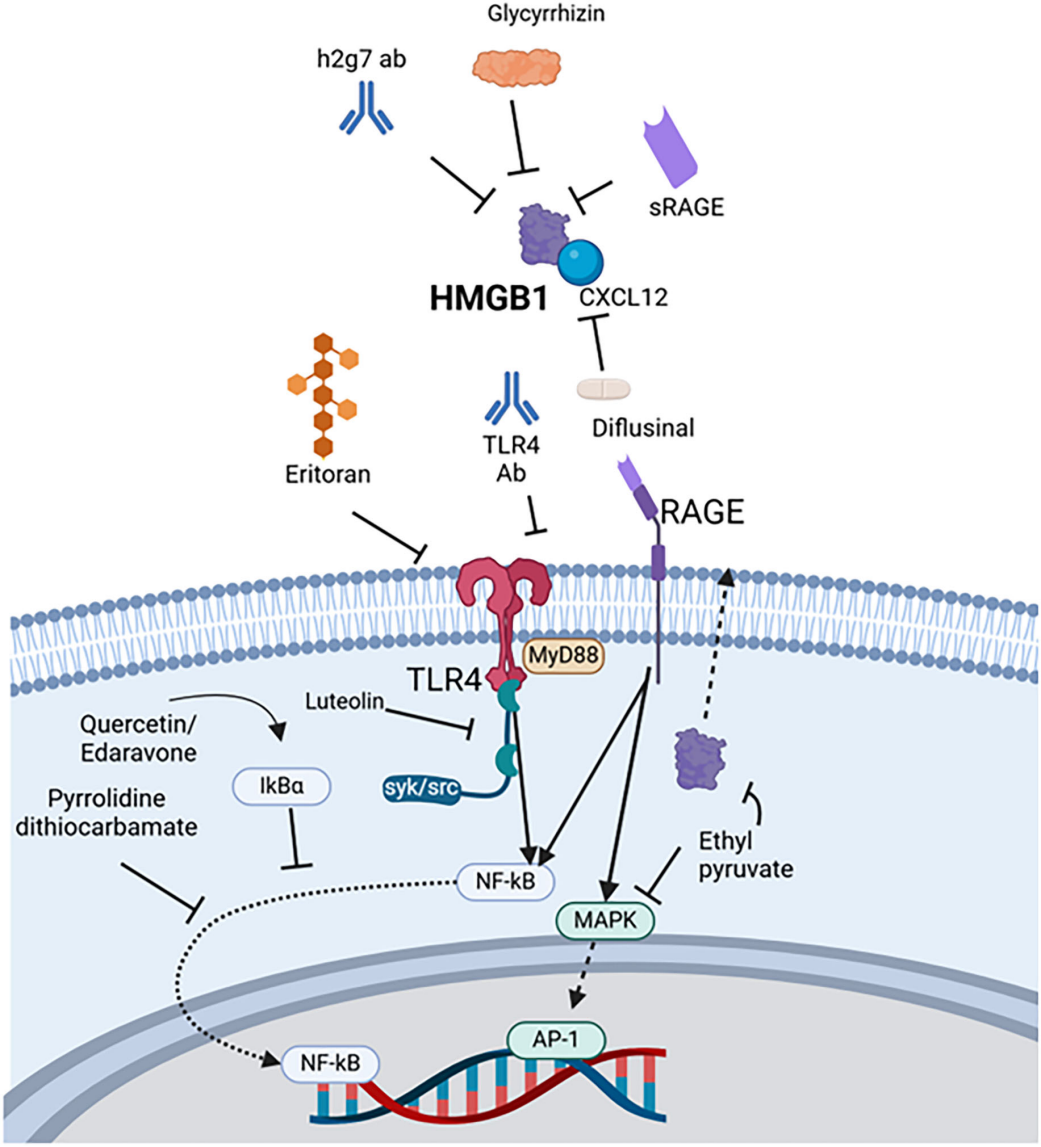
Therapies targeting HMGB1. For HMGB1, h2g7 antibody, glycyrrhizin, and sRAGE all directly bind to the DAMP, whereas diflusinal binds to the HMGB1/CXL12 complex. Eritoran and TLR4 antibody block HMGB1 interaction with this receptor. Luteolin inhibits syk/src activity, and ethyl pyruvate both antagonizes MAPK and prevents HMGB1 release. Quercetin and edaravone preserves and/or recovers IkBα. Pyrrolidine dithiocarbamate inhibits NF-κB translocation to the nucleus. AP-1, Activator protein-1, CXCL12, C-X-C motif chemokine ligand 12, DAP12, DNAX activating protein of 12 kD, HMGB1, High mobility group box 1, MAPK, Mitogen-activated protein kinase, MyD88, Myeloid differentiation primary response protein 88, NF-κB, Nuclear factor kappa-light-chain-enhancer of activated B cells, RAGE, Receptor for advanced glycation end products, sRAGE Soluble receptor for advanced glycation end products, src, Proto-oncogene tyrosine-protein kinase, TLR4, Toll-like receptor 4.

Several agents investigating the therapeutic potential of HMGB1 have been studied (**Figure 3**). HMBG1 neutralizing antibodies treat inflammation by reducing the production of TNFα and IL-6 in sepsis (112). Specifically, h2g7, a partly humanized HMGB1 antibody, improves acetaminophen-induced liver inflammation by decreasing circulating cytokines and lowering ALT (142). Neutralizing antibodies have also ameliorated brain injury secondary to intracerebral hemorrhage induced stroke in rats (81). As a decoy strategy, sRAGE can compete with RAGE to block the HMGB1/RAGE signaling pathway and has been shown to attenuate ischemic damage in stroke (112).

Regarding scavenger molecules, the endogenous protein CD5L is secreted by macrophages and may facilitate clearance of HMGB1 and HSPs in stroke as well as HMGB1 in sepsis according to *in vitro* studies and mouse models (121, 143). Some of the evidence is indirect, however, showing decreases in blood and peritoneal levels with recombinant CD5L treatment or demonstrating weak binding affinity (121, 143). Still, CD5L may represent another method of natural DAMP elimination.

Several other mechanisms to target HMGB1, such as binding to its receptors, have shown anti-inflammatory effects. Eritoran is a derivative of the lipid A component of LPS from *Rhodobacter* sp*haeroides* (123). Eritoran binds to the TLR4-MD2 complex, inhibiting activation by HMGB1 (123). Use of Eritoran attenuated inflammation-based damage in liver I/R and sepsis (123). In addition, antibodies to TLR4 can reduce inflammation by upregulating suppressor of cytokine synthesis 3 (SOCS3) and mammalian target of rapamycin (mTOR), polarizing macrophages to an anti-inflammatory phenotype (122).

Quercetin, a flavonoid, and ethyl pyruvate both protect against reactive oxygen species and inhibit extracellular release of HMGB1 (112, 126). Other antioxidants, such as pyrrolidine dithiocarbamate and edaravone, reduce HMGB1 expression in animal models of pancreatitis and neonatal sepsis, respectively (127, 128). Luteolin has been evaluated through *in vitro* and *in vivo* models (115, 124). Proposed mechanisms include targeting spleen tyrosine kinase (Syk), proto-oncogene tyrosine-protein kinase (Src), and SOCS3 to downregulate cytokine expression mediated by IRF-1, NF-kB, and AP-1 (115). Glycyrrhizin binds HMGB1 and inhibits chemotactic activity, showing efficacy in acute kidney injury, ischemic brain injury, and other diseases (117–120). Sesamin, isolated from sesame oil, reduces HMGB1 levels in sepsis synergistically with other antagonists (134).

Lastly, although FDA approved for rheumatoid arthritis rather than acute pathologies, diflusinal is a derivative of salicylic acid that binds to the HMGB1-CXCL12 heterocomplex and decreases inflammatory cell recruitment (112, 125).

### 6.3 Histones

**Figure 4:**
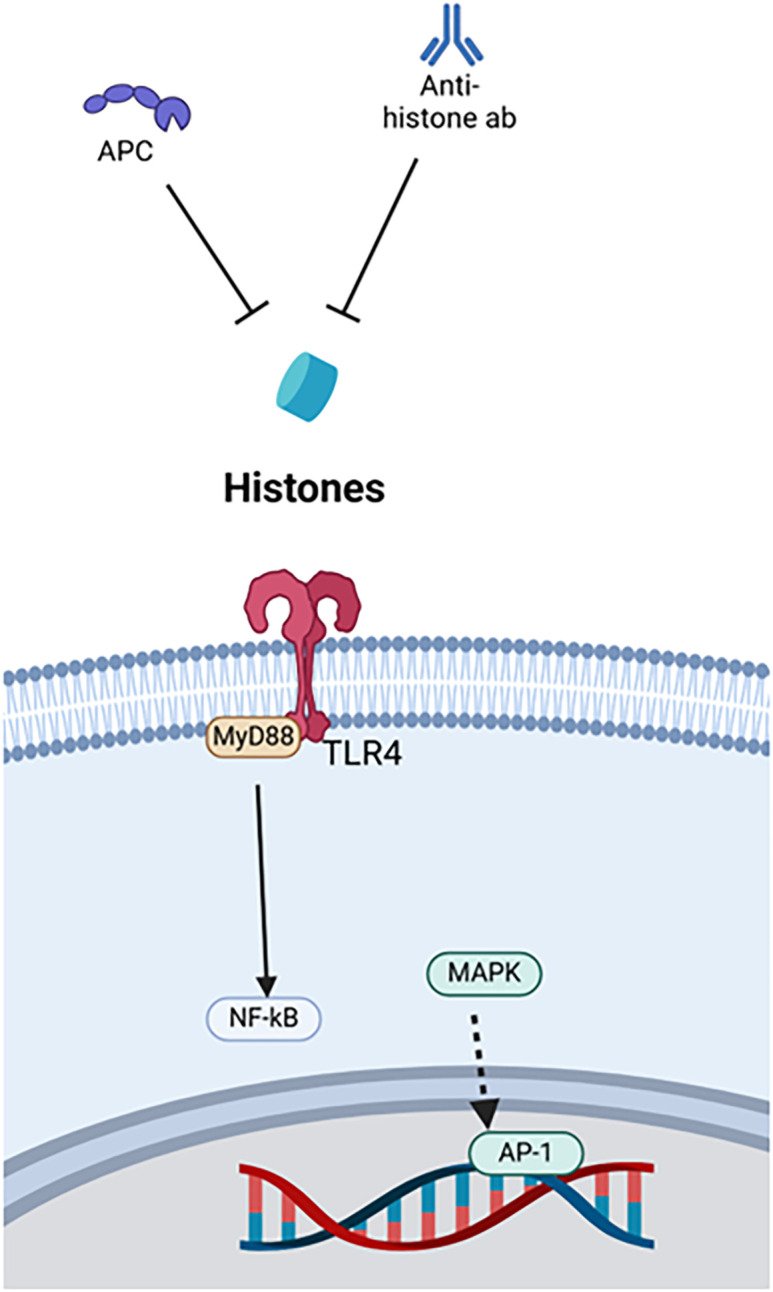
Therapies targeting histones. Histone therapies include APC and anti-histone antibodies, which both bind to the DAMP. APC, Activated protein C, AP-1, Activator protein-1, MAPK, Mitogen-activated protein kinase, MyD88, Myeloid differentiation primary response protein 88, NF-κB, Nuclear factor kappa-light-chain-enhancer of activated B cells, TLR4, Toll-like receptor 4.

In their physiological roles, histones stabilize chromosomes into compact spools of DNA, known as nucleosomes. These nucleosomes are formed by eight subunits, two each of H2A, H2B, H3 and H4 (144). Histones can be modified to regulate DNA transcription (39). In their pathophysiological roles, histones 3 and 4 are prominent CAMPs that act on cell surface receptors TLR4 and TLR2, the endosomal receptor TLR9, and the NLRP3 inflammasome to amplify inflammation (39). These mechanisms have been targeted to treat acute immune dysfunction (**Figure 4**).

One example is anti-H4 antibody. In mouse models of sepsis and endotoxemia, namely CLP and LPS stimulation, extracellular histone levels increase, and survival improves with administration of anti-H4 antibody (129). Another treatment, activated protein C (APC), seems to work synergistically with anti-H4 antibody. APC neutralizes the damaging effects of histone administration in mice, and survival worsens when APC is inhibited during LPS stimulation (129). Beneficial effects of anti-histone antibodies were observed in relation to cardiomyocyte damage during sepsis (65). Anti-histone single-chain variable fragment (ahscFv) antibody was used as a therapy in both *ex vivo* experiments using septic human sera with cardiomyocytes and CLP-induced septic mice. Both models demonstrated decreased injury to cardiac myocytes with the administration of the anti-histone antibody (65). Lastly, *in vitro* models using necrotic EL-4 lymphoblasts to stimulate LPS-primed bone marrow-derived cells showed that both anti-H4 antibody and APC reduced NLRP3 inflammasome activation and neutrophil recruitment via IL-1β. Both agents were used as monotherapies, anti-H4 antibody through blockade and APC through enzymatic degradation, but *in vivo* models were not evaluated (27).

### 6.4 ATP

ATP is produced from cellular respiration in mitochondria and is used as energy (30). After release into the extracellular space, ATP is proinflammatory via the stimulation of purinergic receptors, most notably P2X and P2Y, leading to inflammasome activation (6). This leads to cytokine production, phagocyte recruitment, and activation of the NLRP3 inflammasome, which causes secretion of IL-1β by macrophages (130).

**Figure 5:**
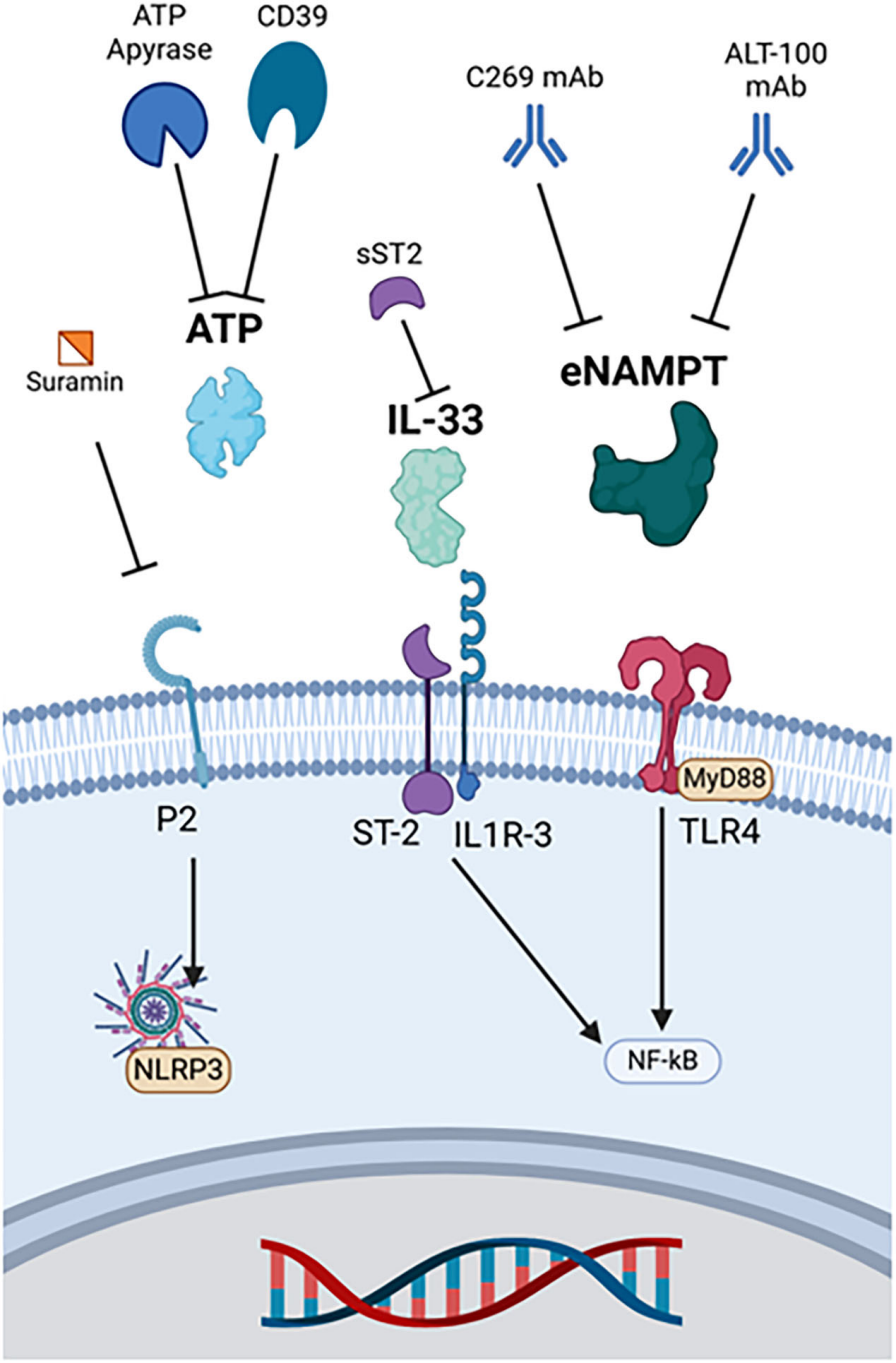
Therapies targeting other major DAMPs. ATP apyrase and CD39 metabolize ATP, and suramin binds to its purinergic receptor to prevent ligand-receptor interactions. Soluble ST2 binds and deactivates IL-33. eNAMPT monoclonal antibodies include C269 and ALT-100. ATP, Adenosine triphosphate, eNAMPT, Extracellular nicotinamide phosphoribosyltransferase, IL-33, Interleukin 33, IL1R-3, Interleukin 1 receptor 3, MyD88, Myeloid differentiation primary response protein 88, NF-κB, Nuclear factor kappa-light-chain-enhancer of activated B cells, NLRP3, Nucleotide-binding domain, leucine-rich repeat, and pyrin domain-containing protein 3, sST2, Soluble suppression of tumorigenicity-2, ST-2, Suppression of tumorigenicity-2, TLR4, Toll-like receptor 4.

Recent studies have shown that targeting extracellular ATP can have important anti-inflammatory effects in sepsis (130). Cytokine production by ATP binding the P2X7 receptor can have severe detrimental effects. Ectonucleoside triphosphate diphosphohydrolase 1 (CD39) expression on macrophages has been shown to dampen ATP-P2X7 signaling by scavenging extracellular ATP, an endogenous example of DAMP breakdown that reduces inflammation (**Figure 5**) (79, 80). *In vivo* studies have shown that CD39 deficient mice had increased levels of IL-10, IL-1β, and TNFα, and blockade of the P2X7 with A438079 has protective effects in acute liver injury (131, 132). In addition, removal of ATP through systemic treatment with apyrase, a nucleotidase CD39 mimic, reduced levels of IL-10, IL-1β, and TNFα in a mouse model of LPS-induced shock (130). Treatment with apyrase can also reduce cytokine levels and improve survival in a CLP murine model of sepsis (131).

Another anti-ATP strategy is inhibition of P2X purinergic receptors, ATP-gated excitatory ion channels (130). The P2X channel has three protein subunits that allow cation passage into the cell upon agonist binding (130). Blocking ATP binding via antagonism of P2X receptors with suramin has potential to inhibit inflammasome activation and reduce inflammation, but experiments have yielded mixed results (130).

### 6.5 IL-33

During homeostasis, IL-33 is expressed in the nucleus of epithelial cells and endothelial cells, binding to histones but with a poorly understood function (28, 50, 133). Knowledge of its role in sepsis continues to develop, whereas its role in acute airway disease is established (50, 133). Once activated and released during stress, IL-33 binds a heterodimeric receptor comprised of ST2 and IL-2 accessory protein, resulting in recruitment of MyD88 and IL-1R-associated kinase for downstream signaling (28).

Studies investigating the therapeutic potential of targeting the IL-33/ST2 axis in sepsis have shown mixed results. sST2, a soluble IL-33 decoy receptor, is significantly elevated in circulation and could be used to inhibit IL-33 (28, 133). While some studies have shown that elevated IL-33 exacerbates tissue damage in the liver, kidney, and lungs of mice via ST2-mediated production of cytokines, other studies have shown that IL-33 ameliorates septic conditions via downregulation of TL4 signaling (28, 133). Sesamin has improved survival and decreased cytokine expression in a CLP mouse model of sepsis (134). These effects were associated with decreased expression of IL-33 and HMGB1 (134). It has been theorized that IL-33 has both beneficial and detrimental effects in sepsis depending on the time course and micro-environment (28, 133). Whether targeting IL-33 is beneficial or detrimental in the setting of acute inflammation remains under investigation.

### 6.6 eNAMPT

NAMPT is an intracellular enzyme involved in nicotinamide adenine dinucleotide synthesis through the conversion of nicotinamide to nicotinamide mononucleotide (22, 145). During ARDS, cells upregulate NAMPT expression, which is excreted extracellularly. eNAMPT functions as a DAMP through ligand-receptor interactions with TLR4, promoting inflammation though upregulating NF-κB activity (22).

Evaluation of eNAMPT-based therapeutics has occurred principally through direct inhibition. Intraperitoneal injection of Alt-100, an eNAMPT monoclonal antibody, reduced intestinal injury and inflammation via inhibition of TLR4 activation and upregulation of transforming growth factor β (TGFβ) in premature Sprague-Dawley pups with necrotizing enterocolitis (21). Protective effects were also found with intravenous administration of Alt-100 in LPS-induced pneumonia models of septic shock and ventilator induced lung injury (VILI) in rats and pigs (135, 146). Overall, Alt-100 can ameliorate systemic inflammation, acute lung injury, and acute kidney injury through neutralization of eNAMPT (135, 147).

Another monoclonal antibody targeting eNAMPT, C269, improved outcomes in a BALB/C murine model of experimental colitis (136). C269 decreased inflammation, including TNFα expression, and improved survival in this experimental model of inflammatory bowel disease, a condition with elevated eNAMPT (136).

## 7 Conclusion and future directions

In this review, we provided a framework to understand DAMPs and presented recent developments regarding therapeutic strategies to neutralize DAMPs. First, we briefly described and classified DAMPs. Having established the therapeutic potential of targeting DAMP release and activity, we then detailed the mechanisms by which major DAMPs are released and subsequently promote inflammation through receptor-ligand interactions. Importantly, we outlined relevant human studies that demonstrate an association between DAMPs and acute inflammatory diseases. Lastly, we analyzed and described the literature studying therapeutic advances toward inhibiting DAMPs to reduce injury and improve outcomes in diseases such as sepsis, ischemia/reperfusion, hemorrhage, and trauma.

The fundamental issue facing acute inflammatory diseases discussed in this review is the disruption of a balanced immune response. While inflammation can facilitate tissue repair and regeneration, acute inflammatory pathologies often amplify the immune system’s reaction to a point of harm rather than benefit (4, 16). The body’s endogenous mechanisms of DAMP clearance and breakdown, such as scavenging proteins (CD5L, MFG-E8) and circulating proteases, are not always sufficient to manage systemic release. Therefore, neutralizing DAMPs represents a valid strategy as an adjunct or even complete therapy, depending on its efficacy. While the complete neutralization of DAMPs may translate to excessive suppression of the immune system, this response is unlikely as evidenced by improved outcomes in DAMP-knockout mice studies (19, 20, 27). Long term consequences are difficult to speculate because animal studies that focus on acute pathologies do not measure or monitor outcomes beyond the early stages and rarely use more than one to three doses of therapeutic to neutralize DAMPs. Even so, mortality in mouse and rat studies worsens without treatment by DAMP neutralizing therapies. Thus, at a minimum, attenuating DAMP activity is beneficial.

Some authors have noted the emergence of LAMPs, such as cholesterol crystals with high fat diets, uric acid in gout, and silica or asbestos from environmental exposure (18, 148). LAMPs perpetuate an inflammatory state, preventing tissue healing and recovery (18). These LAMPs outpace the evolution of our immune system, as modern diseases and exposures manifested after the development of the DAMP response (18). Modern therapies that prolong survival in these previously fatal diseases (hemorrhage, sepsis, ischemia/reperfusion) have likely also outpaced the original adaptive purpose of the DAMP response.

Current therapeutic strategies often focus on neutralizing only one or two DAMPs or their receptors, rather than achieving complete inhibition of all DAMP activity. Given the complexity and interconnectedness of DAMP signaling, completely controlling all molecules released during cellular stress or death may be impractical and unnecessary. Neutralizing a single DAMP or receptor can have cascading effects beyond the initial interaction, potentially explaining the observed benefits of these targeted therapies. Furthermore, incomplete DAMP inhibition may be advantageous, allowing for a balanced immune response rather than excessive immunosuppression. Indeed, some suggest that maintaining DAMP homeostasis, rather than complete ablation, is crucial for proper immune function (16). Nevertheless, therapeutics targeting multiple DAMPs warrant further investigation, as they may offer greater efficacy compared to single-DAMP approaches. Additionally, in larger animal models and clinical trials, exploring multiple dosing regimens or continuous infusions may prove more effective for managing acute pathologies requiring prolonged critical care.

In conclusion, while therapeutically targeting DAMPs shows promise, it also presents challenges. Evaluation of the long-term effects of these therapies as well as dose optimization and toxicity studies are needed. Since most human studies evaluating DAMPs have demonstrated increased levels in the serum, intravenous drugs would likely be most effective. No FDA-approved drugs specifically targeting DAMPs to treat inflammatory diseases have been reported yet. However, a TREM-1 inhibitor, nangibotide, has been evaluated for safety in phase 1 and 2a trials (149, 150). While preliminary doses have not shown significant improvement, higher doses of this TREM-1 inhibitor may improve immune regulation during sepsis (151). Further studies could directly evaluate nangibotide’s ability to inhibit DAMP interaction with TREM-1. In addition, one study on ALT-100 mAb, an antibody for eNAMPT, is in phase 2 trials (ClinicalTrials.gov, NCT05938036). Overall, the study of targeting DAMPs in acute inflammation is promising, and other therapeutics may be on the horizon.

## Glossary

AKT: Protein kinase B
AP-1: Activator protein-1
APC: Activated protein C
ARDS: Acute respiratory distress syndrome
ASC: Apoptosis-associated speck-like protein
ATP: Adenosine triphosphate
CD39: Ectonucleoside triphosphate diphosphohydrolase 1
cGAS: Cyclic guanosine monophosphate-adenosine monophosphate synthase
CLP: Cecal ligation and puncture
CAMP: Chromatin-associated molecular patterns
cfDNA: Cell free DNA
CK2: Casein kinase II
CPEB1: Cytoplasmic polyadenylation element Th-binding protein 1
CREB: Cyclic AMP response element binding protein
CXCL12: C-X-C motif chemokine ligand 12
DAP12: DNAX activating protein of 12 kD
DAMP: Damage associated molecular patterns
eCIRP: Extracellular cold-inducible RNA-binding protein
eNAMPT: Extracellular nicotinamide phosphoribosyltransferase
ER: Endoplasmic reticulum
FMRP: Fragile-X mental retardation protein
GSK3β: Glycogen synthase kinase-3β
HMGB1: High mobility group box 1
HSP: Heat shock protein
ICAM-1: Intracellular adhesion molecule 1
IL-33: Interleukin 33
IL-1R: IL-1 receptor
ICU: Intensive care unit
IRAK: Interleukin-1 receptor-associated kinase
IRF3: Interferon regulatory factor 3
JAK: Janus kinase
JNK: c-Jun N-terminal kinase
LAMP: Lifestyle-associated molecular patterns
LPS: Lipopolysaccharide
MAPK: Mitogen-activated protein kinases
MFG-E8: Milk fat globule-epidermal growth factor VIII
MLKL: Mixed lineage kinase domain-like pseudo kinase
MyD88: Myeloid differentiation primary response protein 88
mTOR: Mammalian target of rapamycin
NAD: Nicotinamide Adenine Dinucleotide
NET: Neutrophil extracellular trap
NF-κB: Nuclear factor kappa-light-chain-enhancer of activated B cells
NLRP3: Nucleotide-binding domain and leucine-rich repeat protein-3
NLS: Nuclear localization sequences
P2: Purinergic 2 Receptor
PBEF: Pre-B-cell colony-enhancing factor
PERK: Protein kinase R-like endoplasmic reticulum kinase
PI3K: Phosphatidylinositol 3-kinase
Poly(A): Poly-adenosine
PRMT-1: Protein arginine methyltransferase 1
PRR: Pattern recognition receptor
RAB: Ras-associated binding protein
RAGE: Receptor for advanced glycation end products
RGD: Arginylglycylaspartic acid
RIPK: Receptor-interacting protein kinases
ROS: Reactive oxygen species
SNARE: Soluble N-ethylmaleimide-sensitive factor activating protein receptor
SOCS3: Suppressor of cytokine synthesis 3
SRC: Proto-oncogene tyrosine-protein kinase
ST2: Suppression of tumorigenicity 2
STAT-1: Signal transducer and activator of transcription 1
STING: Stimulator of interferon genes
SYK: Spleen tyrosine kinase
TBK1: Tank-binding kinase 1
TGFβ: Transforming growth factor β
TIA-1: T-cell intracellular antigen 1
TIAR: TIA-1 related/like protein
TLR: Toll-like receptor
TNFα: Tumor necrosis factor alpha
TREM-1: Triggering receptor expressed on myeloid cells-1
TRIF: TIR-domain-containing adapter-inducing interferon-β
VCAM-1: Vascular cellular adhesion molecule 1
VILI: Ventilator induced lung injury
VNUT: Vesicular nucleotide transporter
